# Cost and cost-effectiveness of soil-transmitted helminth treatment programmes: systematic review and research needs

**DOI:** 10.1186/s13071-015-0885-3

**Published:** 2015-07-03

**Authors:** Hugo C. Turner, James E. Truscott, T. Déirdre Hollingsworth, Alison A. Bettis, Simon J. Brooker, Roy M. Anderson

**Affiliations:** London Centre for Neglected Tropical Disease Research, Department of Infectious Disease Epidemiology, School of Public Health, Faculty of Medicine, St Marys Campus, Imperial College London, Norfolk Place, London, W2 1PG UK; Mathematics Institute, University of Warwick, Coventry, CV4 7AL UK; School of Life Sciences, University of Warwick, Coventry, CV4 7AL UK; Faculty of Infectious and Tropical Diseases, London School of Hygiene and Tropical Medicine, London, UK; Kenya Medical Research Institute, Nairobi, Kenya

**Keywords:** STH, Mass drug administration, Preventive chemotherapy, Cost, Cost-effectiveness, Health economics, NTDs, Systematic review, Economic evaluations

## Abstract

**Background:**

In this time of rapidly expanding mass drug administration (MDA) coverage and the new commitments for soil-transmitted helminth (STH) control, it is essential that resources are allocated in an efficient manner to have the greatest impact. However, many questions remain regarding how best to deliver STH treatment programmes; these include which age-groups should be targeted and how often. To perform further analyses to investigate what the most cost-effective control strategies are in different settings, accurate cost data for targeting different age groups at different treatment frequencies (in a range of settings) are essential.

**Methods:**

Using the electronic databases PubMed, MEDLINE, and ISI Web of Knowledge, we perform a systematic review of costing studies and cost-effectiveness evaluations for potential STH treatment strategies. We use this review to highlight research gaps and outline the key future research needs.

**Results:**

We identified 29 studies reporting costs of STH treatment and 17 studies that investigated its cost-effectiveness. The majority of these pertained to programmes only targeting school-aged children (SAC), with relatively few studies investigating alternative preventive chemotherapy (PCT) treatment strategies. The methods of cost data collection, analysis and reporting were highly variable among the different studies. Only four of the costing studies were found to have high applicability for use in forthcoming economic evaluations. There are also very few studies quantifying the costs of increasing the treatment frequency.

**Conclusions:**

The absence of cost data and inconsistencies in the collection and analysis methods constitutes a major research gap for STH control. Detailed and accurate costs of targeting different age groups or increasing treatment frequency will be essential to formulate cost-effective public health policy. Defining the most cost-effective control strategies in different settings is of high significance during this period of expanding MDA coverage and new resource commitments for STH control.

**Electronic supplementary material:**

The online version of this article (doi:10.1186/s13071-015-0885-3) contains supplementary material, which is available to authorized users.

## Review

The primary control strategy for soil-transmitted helminths (STH) is regular periodic mass drug administration (MDA), also called preventive chemotherapy (PCT), targeting Pre-School Aged Children (Pre-SAC) and School Aged Children (SAC) [1, 2]. These control programmes originally depended on vertical programs in which mobile teams visited schools or communities to distribute the drugs [3, 4]. Nowadays, they are predominantly centred on school-based delivery systems, utilising teachers and other school officials [3, 4]. This delivery method enables the programmes to be linked in to the school educational system [3], which has been shown to be both highly cost-effective, and an effective method to reach children in poor rural areas [3, 4].

The World Health Organization (WHO) recommends that MDA programmes prioritise SAC, but also recommends the treatment of Pre-SAC, women of child-bearing age, and adults in certain high risk occupations (such as tea-pickers and miners) [1]. In the majority of endemic areas, treatment is given annually, but in areas of intense transmission (defined as a prevalence of any STH greater than 50 % in SAC), the WHO recommends that the treatment frequency is increased to at least twice a year (depending on resource availability) [1]. In areas where lymphatic filariasis (LF) is endemic, the whole community may be treated through LF control programmes.

There is currently a period of intensifying MDA coverage and new resource commitments for STH control [5, 6, 7]. The WHO and the London declaration on NTDs have set goals to scale up MDA, so that by 2020, 75 % of the Pre-SAC and SAC in need, will be treated regularly [6, 7]. However, many questions remain regarding how best to deliver STH treatment programmes to achieve the greatest impact; these include which age-groups should be targeted and how often.

Mathematical models have illustrated that the optimum target age-group is highly dependent on the age distribution of the different STH species [8–10]. For instance, in areas with a medium-high prevalence of hookworm (for which unlike the other STHs the infection intensity peaks in adulthood as opposed to childhood/adolescence [11–14]) it will likely be necessary to expand treatment to include adults; particularly in the context of breaking transmission [8–10]. Consequently, the optimum treatment strategy will be highly specific to the local epidemiology.

To perform further analyses to investigate what the most cost-effective control strategies are in different settings, accurate cost data for targeting different age groups at different treatment frequencies (in a range of settings) are essential. This will be crucial to inform the most efficient use of the expanding resources for STH control [5].

In this paper, we identify, summarise and analyse the range of costing studies and cost-effectiveness analyses which have been performed for the different potential STH treatment strategies. We then outline the crucial gaps in knowledge which are essential to evaluate changes in strategies beyond the current policies.

### Literature search

**Fig. 1:**
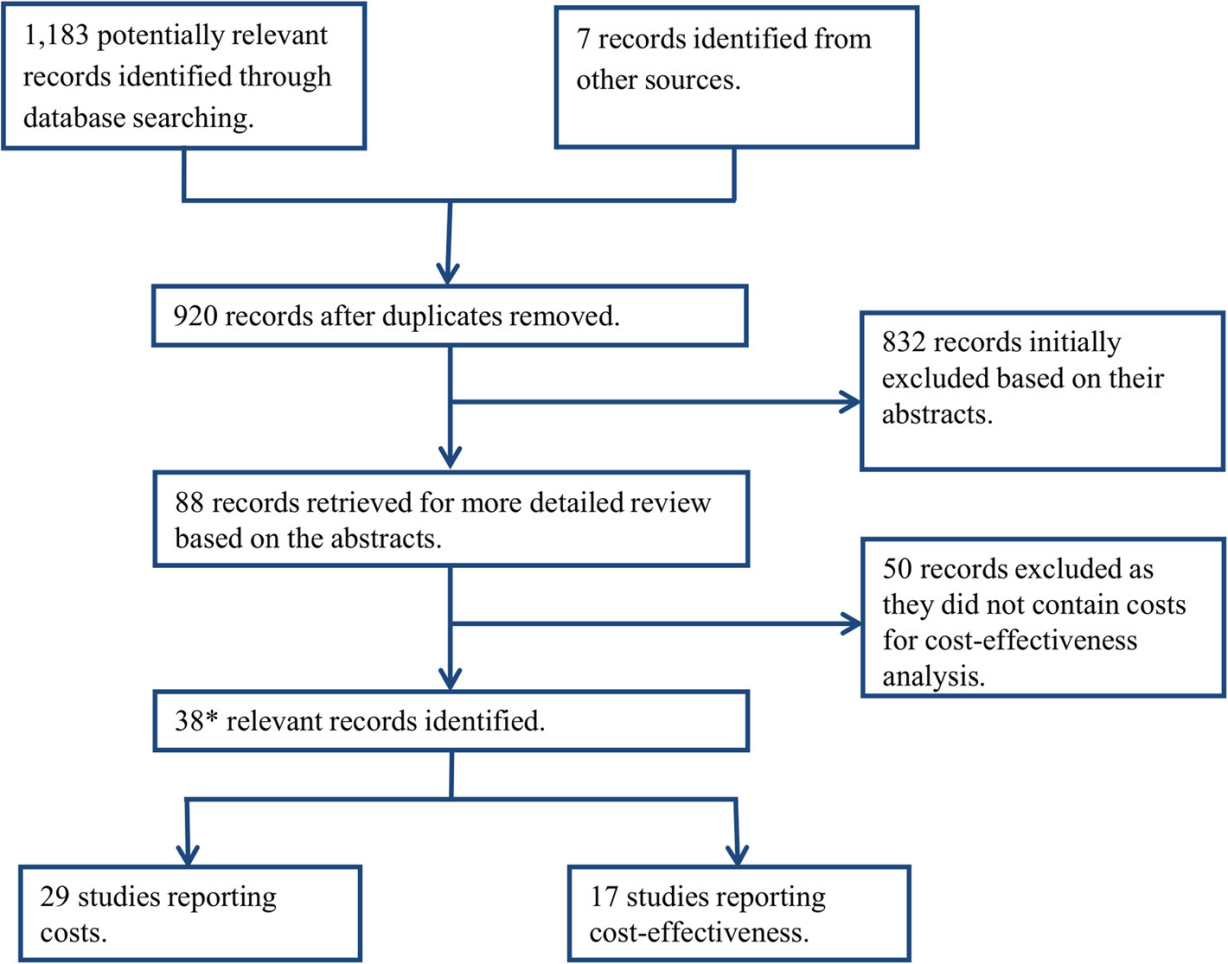
Decision tree outlining the inclusion and exclusion of the identified studies; * Several studies reported both costs and cost-effectiveness estimates. *A PRISMA* checklist is provided in Additional file 2

Systematic searches were performed in October 2014 using the electronic databases PubMed, MEDLINE, and ISI Web of Knowledge, using the possible variants of the terms “Soil-transmitted helminths (including variants on helminth and individual species names), cost(s), cost analysis, economics, economic evaluation, cost benefit, cost-effectiveness” (see Additional files 1 and 2). We imposed no language or date restrictions and the retrieved studies were searched for articles that were not identified in our database searches. Attempts were made to access reports/policy documents not included in the electronic databases. The literature selection process is outlined in Fig. 1. All papers that provided cost estimates for the delivery of STH treatment were considered relevant, even if they did not fully satisfy the criteria of a costing study [15]. The identified studies containing costs were stratified by the target age group, method of delivery and treatment frequency.

The costing studies were grouped into three categories, low, medium and high (Table 1), reflecting their applicability for use in necessary forthcoming economic evaluations of alternative STH treatment strategies. This grouping was based on three factors; 1) whether the cost year and currency exchange rates were clearly stated, 2) economic costs collected, and 3) detailed itemised costs reported for the STH control component of the programme (i.e. no major costs sources were excluded). Those that provided/did all three were grouped into high, two into medium, and only one or none into low.

**Table 1:** Overview of the identified costs

Study	Country	Target of intervention	Primary distribution method	Age-groups targeted	Treatment frequency	Perspective explicitly stated	Year of price	Currency	Economic costs collected	Costs itemised	Results
*High*											
[24]	Uganda	STH and Schistosomiasis	School-based	SAC	Annual	Y (Service provider)	2005	US$	Y	Y	The overall economic cost per child treated in the six districts was US$0.54, which ranged between the districts from US$0.41 to US$0.91 (delivery costs: US$0.19–0.69). The overall financial cost per child treated was US$0.39.
[25]	Haiti	STH and Lymphatic filariasis	Combination	Mass treatment (all persons greater than two years of age)	Annual	Y (Service provider)	2008–2009	US$	Y	Y	The economic cost was US$0.64 per person treated, which included the value of the donated drugs. The programme cost (which excluded the value of the donated drugs) was US$0.42 per person treated.
[26]	Niger	STH and Schistosomiasis	Combination	SAC and targeted adults	Annual	N	2005	US$	Y	Y	The total economic delivery cost of the school-based and community-based treatment was US$0.76, and US$0.46 respectively. Including only the programme costs and the values change to US$0.47 and US$0.41 respectively. The average drug (albendazole and praziquantel) cost was US$0.28 per treatment; not clear which results included the drugs costs.
[27]	Niger	STH, Schistosomiasis*,* Lymphatic filariasis, and Trachoma	Combination	SAC and adults (not clear if Pre-SAC were treated)	Annual	N	2009	US$	Y	Y	The average economic cost of integrated preventive chemotherapy was US$0.19 (excluding drug costs). The average financial cost per treatment of the vertical schistosomiasis and STH programme (before the NTD programmes integrated) was US$0.10.
*Medium*											
[19]	Lao PDR	STH within an immunisation and vitamin A supplementation campaign	Child Health Days	Pre SAC and women of child-bearing age (SAC were targeted though the national deworming campaign)	Annual	N	2009	US$	Y	Y	The incremental cost of adding deworming into the national immunisation campaign was US$0.03 per treatment (delivery costs: US$0.007). This is compared to US$0.23 per treatment for the vertical national school-based deworming campaign (targeting SAC).
[20]	Nigeria	STH, Schistosomiasis, Lymphatic filariasis, and Onchocerciasis	Community drug distributers (CDDs)	SAC for praziquantel and SAC and adults for ivermectin/albendazole	Annual	Y (Service provider)	2008–2009	US$	N	Y	In 2008, eight local government areas received a single round of ivermectin and albendazole followed at least one week later by praziquantel to SAC. The following year, a single round of triple drug administration was given. When using the latter the programmatic costs for MDA (not including drug and overhead costs), were reduced by 41.1 % (from US$0.078 to US$0.046 per treatment).
[22]	Ethiopia	STH	Child Health Days	Pre-SAC	One round	N	2006	US$	Y	Partial	The average cost per child reached by the Child Health Day programme was US$0.56 (per round) of which deworming was estimated to represent 29 % of the cost (US$0.162).
[23]	Uganda	STH within an vitamin A supplementation campaign	Child Health Days	SAC and Pre-SAC	One round	Y (Service provider)	2010	US$	Y	Partial^#^	The average cost per child reached by the Child Health Day programme was US$0.22 (per round) – including the cost of vitamin A.
											*# Although detailed itemised costs were provided they pertained to the Child Health Day programme as a whole (the purpose of that study) and not the deworming arm.*
[21]	Based on data from Montserrat	STH	Mobile teams	Mass treatment	Not applicable	Y (Service provider)	1988	US$	N	Y	Presented in a cost menu.
[17]	Tanzania	STH	School-based	SAC	One round	Y (Service provider)	1996	US$	Y	Partial*†*	See [16]
[16]	Ghana and Tanzania	STH	School-based	SAC	One round	Y (Service provider)	1996	US$	Y	Partial*†*	The economic cost per treatment in Ghana, and Tanzania was US$0.27, and US$0.26 (delivery: US$0.07, and US$0.06), respectively. The financial cost per person treated in Ghana, and Tanzania was US$0.24, and US$0.023 (delivery: US$0.04, and US$0.03), respectively.
											*† Note that the results are artificially low because they did not include the external costs of the UK-based coordinating centre* [39]*.*
*Low*											
[37]	Seychelles	STH and other intestinal parasitic infections	Schools and (crèches for 3–5 year olds)	SAC and Pre-SAC (3–5 year olds)	Four monthly	N	1993–1994	US$	N	Y	The financial cost of the programme in 1994 was estimated to be US$0.40 per person treated; unclear if the start-up costs from 1993 were included or if this is a cost per round or per year.
[29]	India	STH (primarily *Ascaris*)	Mobile teams	Pre-SAC	Biannual (six monthly)	Y (Patient)	1995–1997	Indian Rupees (₹)	N	N	The incremental financial cost of treating 5,000 Pre-SAC with six monthly albendazole for two years was ₹122,091 (including the drug cost of ₹20 per dose).
[30]	Vietnam	STH (within a weekly iron-folic acid supplementation campaign)	Village health workers	Non-pregnant women of child-bearing age	four monthly in the first year and six monthly thereafter	Partial	2010	US$	N	Partial	The yearly financial cost of the programme was US$0.76 per woman treated; including the cost of weekly iron supplementation.
[74]	Egypt	STH, Schistosomiasis and other intestinal parasitic infections	Mobile teams	SAC	Annual	N	2000	US$	N	Partial	The incremental financial cost of STH control was US$0.07 per treatment (delivery costs: US$0.03), when integrated into the national schistosomiasis control programme.
[33]	Burundi	STH, Schistosomiasis and other intestinal parasitic infections	Mobile teams (via the school)	SAC (selective treatment)	Annual	N	1984–1992	US$	N	Partial	The financial cost per person protected in 1984–1985, 1989–1990, and 1991–1992 was US$2.7, US$1.2, and US$0.70, respectively. The reported costs per treatment related to only schistosomiasis.
[75]	Burkina Faso	STH and Schistosomiasis	Combination	SAC	One round	N	2004–2005	US$	N	Y	The financial cost per child treated was US$0.308 for the school-based component and US$0.33 for the community-based component (delivery: US$0.084, US$0.107 respectively).
[39]	Based on data from Tanzania	STH and Schistosomiasis	School-based	SAC	Not applicable	N	Not clear	US$	N	Y	Presented in a cost menu [39].
[31]	Nigeria	*Ascaris*	Mobile teams	Varied: A) selective treatment (treating the 20 % most heavily infected), B) targeted treatment to Pre-SAC and SAC and C) mass treatment to all (excluding <1 and pregnant women)	Three monthly	N	1989	Naira	N	Partial	The total financial costs (and delivery costs) were; A) Selective treatment: 2,491 (12,414), B) Targeted treatment: 3,956 (3,550), C) Mass treatment: 4,701 (3,809).
											(Total costs are shown as it is misleading to compare the cost per treatment for a selective treatment strategy to that of mass/targeted treatment.)
[18]	Uganda	STH	School-based	SAC	Annual	Y (Service provider)	2004	US$	N	Partial*‡*	The estimated financial cost per treatment in the four districts ranged from US$0.063 to S$0.105 (delivery costs: US$0.04 to US$0.08). *‡ These cost estimates do not include the start-up costs or those incurred at the central level.*
[32]	Bangladesh	STH	Mobile teams	First dose mass (i.e. children and household members) other doses just children (2–8 years old)	Varied: See legend *(Treatment frequency Note 1)*	Y (Service provider)	Not clear	Takas (৳)	N	Partial	Project cost per household: A) ৳301 B) ৳1,897 C) ৳332 D) ৳1,909
[28]	Nepal	STH within an vitamin A supplementation campaign	Child Health Days	Pre-SAC	Biannual	*NA*	*NA*	US$	*NA*	*NA*	An additional US$80,000 (4 % of the total cost of the vitamin A campaign) covered the cost of biannual deworming).
[62]	Zanzibar	STH and Schistosomiasis	School-based (“sibling approach*”)	Non-enrolled SAC	One round	N	2000	US$	N	N	The costs linked to drug transport, training and drug administration were not increased by the inclusion of non-enrolled children. Therefore, the additional financial cost of including non-enrolled SAC using the sibling approach” consisted only of the extra drugs treatments needed. It was noted that a negligible additional cost may be incurred for storage of leftover drugs.
											*Enrolled children invited tell parents, siblings and friends of school-age when the next deworming day is.
[76]	Myanmar	STH	School-based	SAC	One round	N	Not clear	US$	N	Y	A crude calculation estimated that the financial cost per treatment was approximately US$0.05 (delivery: US$0.03).
[77]	Vietnam	STH	School-based	SAC	Annual	N	Not clear	US$	N	Y	The financial costs per treatment in 2000–2001, 2002–2003, and 2005–2006 were US$0.71, US$0.11, and US$0.03 (delivery: US$0.683, US$0.0857 and US$0.0128) respectively.
[78]	Yemen	STH and Schistosomiasis	Combination (school-based for SAC and CDDs/health workers for adults and non-enrolled SAC)	SAC and adults	Annual	N	2008–2009	US$	N	Y	The financial cost per person treated in 2008, and 2009 was US$0.79, and US$0.66 (delivery: US$0.44 and US$0.37), respectively.
[36]	Lao PDR	STH	School-based	SAC	Biannual	N	2007	US$	N	Y	In the provinces treating twice a year the financial cost (capital costs not annualised) was US$0.23 per child per year, while in provinces treating once a year the cost was US$0.17 per child per year.
[35]	Cambodia	STH	School-based	SAC	Biannual	N	2003–2004	US$	N	Y	The financial cost per treatment in 2003 and 2004 was US$0.122, and US$0.057 (delivery: US$0.096 and US$0.033), respectively.
[34]	Notional	STH and Schistosomiasis	Mobile teams (via the school)	SAC	Annual	N	Not clear	US$	N	N	Treating for ten years would cost between US$8 and US$18 per child (US$0.8- US$1.8 per year). (Assumes that four treatments of praziquantel and eight of albendazole are given in the ten year period.

The costing studies were grouped into three categories, low, medium and high, reflecting their applicability for use in forthcoming economic evaluations. This grouping was based on three factors; 1) whether the cost year and currency exchange rates were clearly stated, 2) economic costs collected, and 3) detailed itemised costs reported for the STH control component of the programme (*i.e.* no major costs sources were excluded). Those that provided/did all three were grouped into high, two into medium, and only one or none into low. CDDs; Community drug distributers, Pre-SAC; Pre-school aged children, SAC; School aged children. School-based delivery systems were defined as those utilising teachers and other school officials (not just distributing the drugs at the school NA: Not available. Treatment frequency Note 1*:* Varied; A) Chemotherapy to all household members at the start of the study, B) same as Group A, but with regular health education, C) Chemotherapy to all household members and subsequent six monthly chemotherapy to all children, D) same as Group C but also with regular health education

## Results

### Reported costs of STH treatment

We identified 29 studies that reported costs associated with treatment for STH control (Table 1). A summary of the studies is presented in Table 1 (broken down by the primary distribution method, age-groups targeted, treatment frequency, the economic features of the study, and the results). The majority of studies (18 of 29 (62 %)) were judged to have a low applicability for use in upcoming economic evaluations. Many of these were macro-costings of the financial costs (Box 1), which do not account for several of the key aspects of the treatment programmes (such as the economic value of the time volunteered by teachers/community drug distributors (CDDs) and donated items). Furthermore, several of the reported costs were artificially low because they did not include the costs of their UK-based coordinating centre [16, 17] or only collected data at certain programmatic levels (often not accounting for the costs borne at the national level) [18]. This must be taken into account when comparing the reported costs between different studies, or when using the data for subsequent cost-effectiveness analysis.

**Box 1: Glossary**

**Table:** 

Economic costs: These include estimates of the monetary value of goods/services for which there is no financial transaction or when the price of a specific good does not reflect the cost of using it productively elsewhere. Examples of resources which have no financial costs but do have important economic costs are the ‘free’ use of building space provided by Ministries of Health, and the time devoted to MDA by community drug distributors (CDDs) and teachers. Economic costs are important when considering issues related to the sustainability and replicability of interventions.
Economies of scale: The reduction in the average cost per unit resulting from increased production/output: in this case the reduction in the cost per treatment as a result of increasing the number treated.
Economies of scope: The reduction in the average cost per unit resulting from producing two or more products at once: in this case the reduction in the cost per treatment, when delivering more than one intervention at once (i.e. integrated control programmes).
Financial costs: Those were a monetary transaction has taken place for the purchase of a resource.
Fixed costs: Costs that are not dependent on the amount of output: in this case costs that are not dependent on the number treated.
Macro-costing: Macro-costings (also known as gross costing or top-down costing) identify cost components at a highly aggregated level, often only allocating a total budget to specific programme activities.
Micro-costing: Micro-costing studies (also known as down-up costing) collect detailed data on resources utilized and the value of those resources.
Perspective: The perspective of the analysis determines which costs are included i.e. the patients, service providers or the society as a whole.
Sensitivity analysis: A sensitivity analysis is a repeat of the primary analysis, substituting alternative decisions or ranges of values for decisions that were arbitrary or unclear.
Variable costs: Costs which vary in proportion to the quantity of output: in this case costs that are dependent on the number treated.

Seven studies were found to have medium [16, 17, 19–23] and only four high [24–27] applicability for the use in economic evaluations. It should be noted that the more in-depth costing studies [24–27] – which also collected economic costs – reported notably higher MDA delivery costs (Table 1).

The method of cost data collection, analysis and reporting was highly variable among the different studies, with many not providing itemised costs stratified by programme activities (Table 1). Several studies reported costs for programmes that were not targeting STH alone making it difficult to separate out the incremental costs for only treating STH. This adds notable complexity to comparing the costs of different treatment strategies. Furthermore, in many cases the cost year/currency exchange rates were not explicitly stated, making it problematic to adjust the results of the different studies to account for inflation, allowing valid comparison. These issues are discussed more in the research needs section below (*Cost data collection and analysis methods).*

**Fig. 2:**
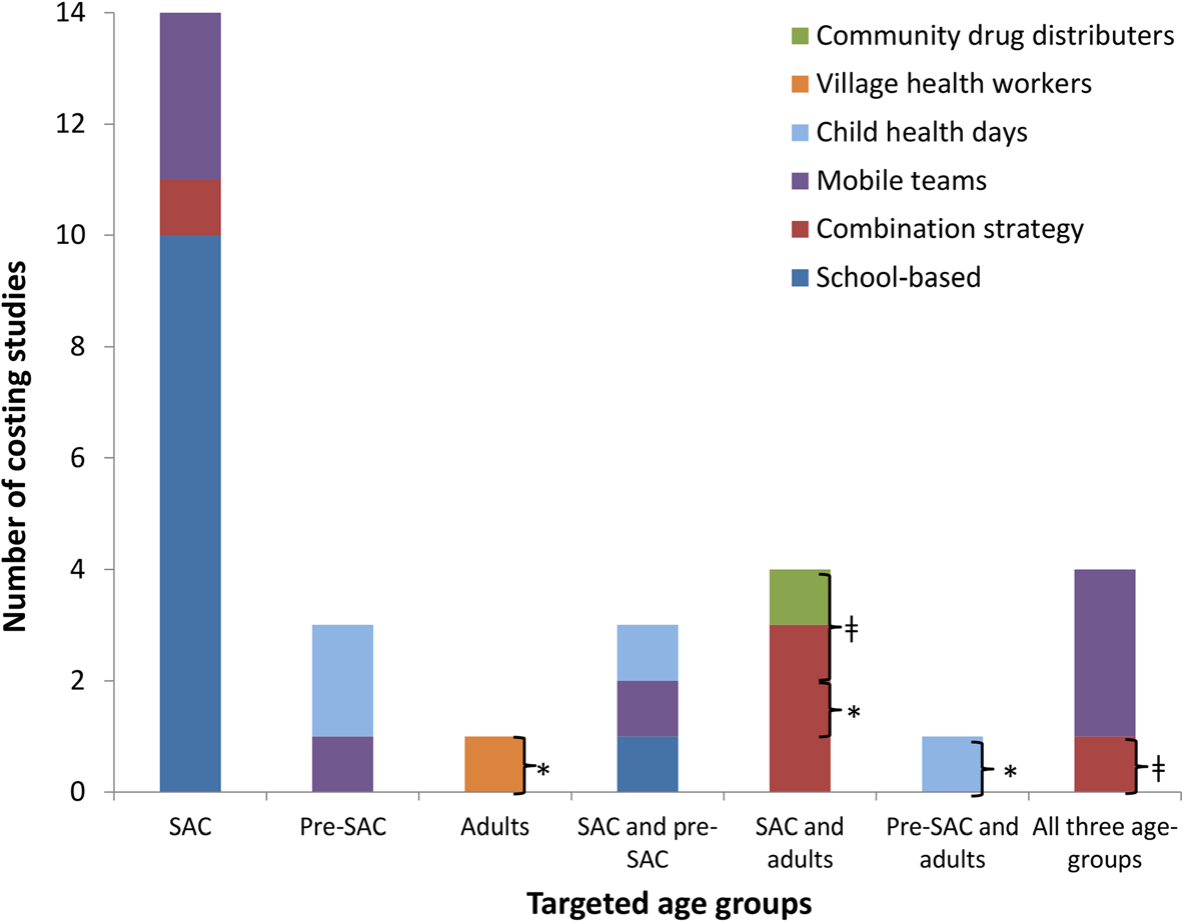
The number of identified costs for STH treatment stratified by the target age group and the method of distribution. Pre-SAC; Pre-school aged children, SAC; School aged children. School-based delivery systems were defined as those utilising teachers and other school officials (not just distributing the drugs at the school). A combination strategy was defined as using both the school system and community drug distributers (CDDs). Some studies were counted more than once, as the target population was varied within the study. * Targeted adults (such as those in at risk occupations); ǂ Programme also targeting lymphatic filariasis (LF)

Due to the inconsistencies in both the collection and analysis methods, as well as the potential differences between different countries/health systems, it was not possible to draw firm conclusions regarding the costs of the different treatment strategies. However, looking at the number of studies stratified by the target age group, method of distribution, and treatment frequency, reveals some important insights regarding the current gaps in the literature (Fig. 2).

#### Target population and method of distribution

**Fig. 3:**
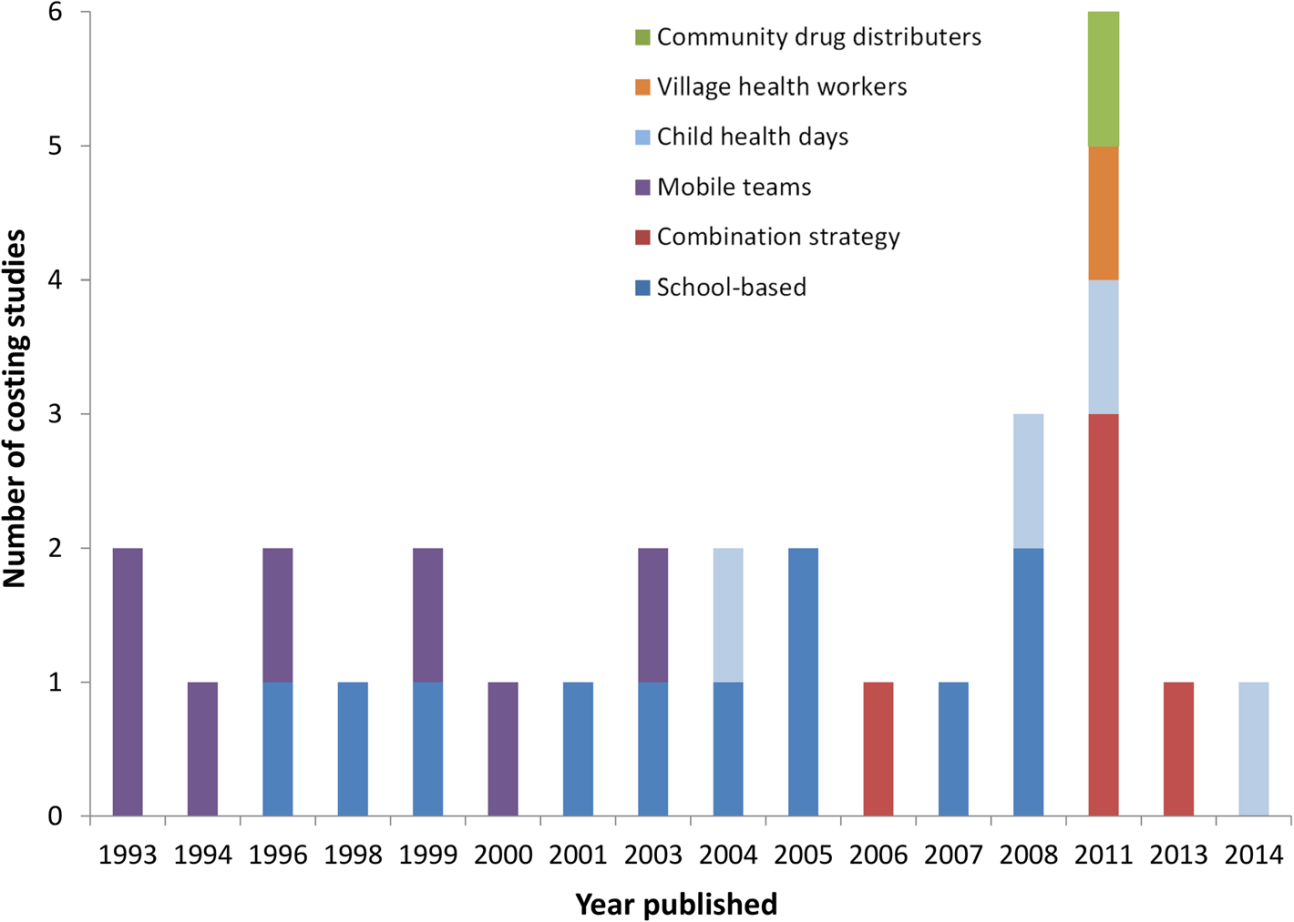
Distribution of the published costing studies over time stratified by the method of distribution. School-based delivery systems were defined as those utilising teachers and other school officials (not just distributing the drugs at the school). A combination strategy was defined as those using both the school system and community drug distributers (CDDs)

The clear majority of the identified costing studies were related to programmes targeting SAC through the school system (Fig. 2). The older studies were more likely to pertain to the use of mobile teams (Fig. 3). However, this method has gradually been replaced by school or community-based delivery systems (Fig. 3). In Africa, a combination strategy was often used, using both the school system to reach enrolled SAC, and CDDs/health workers to reach un-enrolled SAC and/or other age groups in the community.

The three studies identified for programmes targeting only Pre-SAC integrated their distribution either into Child Health Days [22, 28] or used mobile teams [29]. Two studies were identified that investigated the cost of treating women of reproductive age within existing immunisation and vitamin A or iron supplementation campaigns [19, 30]. These studies either integrated their distribution into Child Health Days (also targeting Pre-SAC) [19] or used village health workers [30].

Only 11 studies were found which reported costs pertaining to programmes targeting more than one age group (Pre-SAC, SAC and adults) (Fig. 2). Nine studies reported costs for programmes which included the treatment of adults. However, these were either for programmes integrated with LF control [20, 25, 27], programmes targeting only specific groups of adults [19, 26, 30] or programmes based on the use of mobile teams to distribute the drugs [21, 31, 32]. This is important as the treatment of adults will be essential in many areas in order to break transmission [8–10].

Two studies reported the costs of selective treatment, where people were screened and only treated if infected or heavily infected [31, 33]. Even though this approach uses less drugs than age-targeted or mass treatment, the requirement of having to screen for infection before treatment results in the programme being relatively costly; Holland *et al.* [31] found that selective treatment was three times more expensive than targeted control, although it should be noted that both arms of the study used mobile teams. This is supported by a theoretical analysis of helminth control (not specific to STH) presented by Warren *et al.* [34], which found that selective treatment was both less effective and more costly then mass and targeted treatment strategies. However, it should be noted that both of these studies pertained to the use of mobile teams (which now have mostly been replaced by other delivery systems (Fig. 3)). Though these findings are very likely to be robust within the current school/community-based delivery systems, selective treatment may need to be reassessed if new and more rapid diagnostics are developed.

#### Treatment frequency

**Fig. 4:**
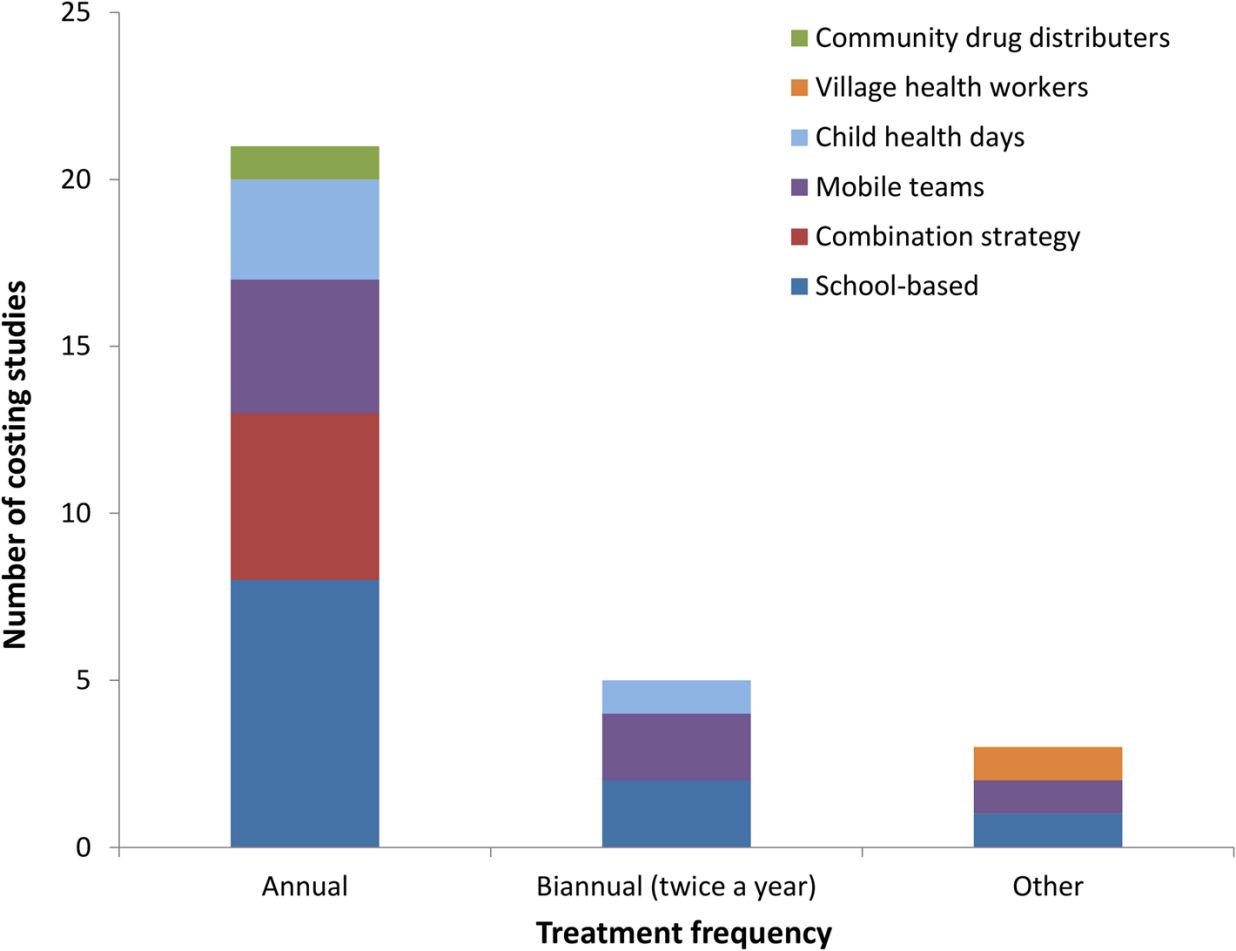
The number of identified costs for STH control, stratified by treatment frequency and the method of distribution. School-based delivery systems were defined as those utilising teachers and other school officials (not just distributing the drugs at the school). A combination strategy was defined as those using both the school system and community drug distributers (CDDs). Studies that just reported the costs of one treatment round were classed as annual. Some studies were counted more than once as the treatment frequency was varied within the study

The majority of the studies related to the use of an annual treatment strategy or just investigated one treatment round (Fig. 4). Only two studies were found which reported the costs associated with biannual treatment within a school-based programme [35, 36]; however, these only reported financial costs, and how increasing the treatment frequency may influence the economic costs (Box 1) has not been investigated. The handful of costing studies found for higher treatment frequencies used mobile teams (which are now rarely used (Fig. 3)) or health workers to distribute the drugs (and generally at a small scale) [30, 31, 37].

#### Integration (economies of scope) and economies of scale

As discussed above, many of the reported costs were for control programmes targeting more than one NTD, or deworming integrated within other control programmes (such as vitamin A supplementation campaigns) (Table 1). It was not always possible to separate out the costs for treating STH, making it difficult to compare the reported costs of different STH treatment strategies.

Evans *et al.* [20] and Leslie *et al.* [27], found that integrating PCT programmes across the NTDs produced economies of scope (Box 1), reducing the overall cost by 16 % to 40 % (this is a comparison of the overall cost of an integrated programme versus the total cost of using separate vertical programmes). This highlights the critical need to consider the local context of the NTD control programmes when comparing the reported costs of MDA, or when using the costs for subsequent cost-effectiveness analysis.

**Fig. 5:**
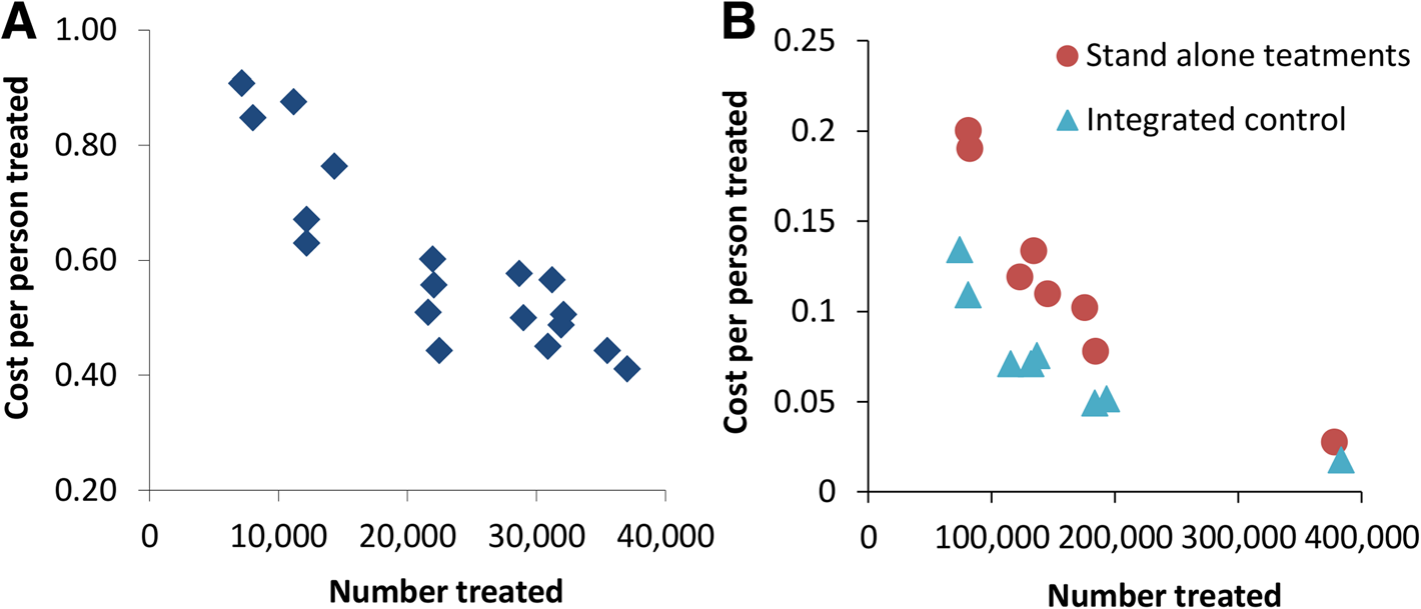
Observed economies of scale associated with mass drug administration (MDA). Data from **a**: Brooker et al. [24], **b**: Evans et al. [20]

Two studies [20, 24] observed that the cost per treatment notably decreased with increasing numbers treated i.e. economies of scale (Fig. 5)*.* This occurs because some of the costs associated with MDA delivery are fixed (i.e. do not depend on the number treated), and therefore increasing the number treated reduces the average fixed cost per treatment. These economies of scale may account for a notable degree of the observed variation in the delivery costs of STH treatment (Table 1) and need to be carefully considered when comparing the costs of different strategies [24, 38]. For example, the economies of scale associated with school-based treatment programmes will likely be notably different to those of programmes using mobile teams (which would probably have a higher variable cost per treatment (Box 1)).

#### Adopted costing perspective

The perspective of the analysis determines which costs are included i.e. the patients, service providers or the society as a whole. In the majority of the studies the perspective was not explicitly stated (or justified), though it was possible to infer from which costs were included that in almost all of the studies the perspective used was that of the service provider i.e. the control programme. One study [29] was found that collected costs from the perspective of the families (payers) – in this case only the cost of the drug was reported as being relevant, which may not be generalisable to settings where patients have to travel to get access to treatment.

#### Issues of time (annualisation and discounting)

There where notable inconsistencies in whether or not studies annualised the costs of capital resources; this is the process whereby the gross cost of capital resources (i.e. those which last longer than a year) are spread over their average useful lifetime, to arrive at an average yearly cost. This may produce a disparity in the reported costs between different studies, particularly for control programmes in their first year, when many of the capital resources will be purchased. Furthermore, assumptions regarding the useful lifetime of capital resources and the discount rate used were rarely stated or subjected to sensitivity analysis.

#### Drug costs

We observed a notable variation in the reported drug costs between the different studies (Table 1). This is in part because the drugs have become cheaper over time [39], at times were donated (and therefore would only be included in the full economic cost), and due to variations in which other drugs were administered/purchased within the same programme. A more in-depth description of the drug costs and how they have changed over time is presented in [39]. More recently Montresor *et al.* [40] estimated the median price for a container of 1,000 tablets of albendazole (400 mg) was US$18.1 (range US$15.1–28), giving an average unit cost of US$0.018. A similar cost was estimated for mebendazole (500 mg) with the median price for a container of 1,000 tablets being US$19.1 (range US$11.9–27.6). The cost of international transport and custom clearance has been estimated to be an additional 10 % of the total value of the drug [40].

### Reported cost-effectiveness of STH treatment

We identified 17 studies that investigated the cost-effectiveness of STH treatment (Table 2). The majority of the cost-effectiveness estimates pertained to interventions targeting only SAC; which were mostly found to be within the range of being highly cost-effective based on the World Bank thresholds [41]. Though it should be acknowledged that the methodology and key assumptions were often unclear, and surrounded by notable uncertainty– particularly for the estimates in terms of US$ per DALY averted [42, 43].

**Table 2:** Summary of the identified cost-effectiveness estimates

Study	Question	Target of intervention	Target age group/ primary distribution method/ treatment frequency	Effects	Primary conclusions	Source of the costs
*Empirical studies*						
[29]	Cost-effectiveness of albendazole for preventing stunting in Pre-SAC.	STH (primarily *Ascaris*)	• Pre-SAC	• Prevention of stunting	Six monthly albendazole reduces the risk of stunting in Pre-SAC with only a small increase in the expenditure on health care from the payer’s perspective (₹543 Indian Rupees for each case of stunting prevented).	Same study
			• Mobile teams			
			• Biannual (six monthly)			
[24]	Cost-effectiveness of nationwide school-based helminth control in Uganda.	STH and Schistosomiasis	• SAC	• Anaemia cases averted	The cost per anaemia case averted was estimated to range from US$1.70–9.51 (depending on the number treated within the different districts (see Table 1)).	Same study
			• School-based treatment			
			• Annual			
[30]	The cost-effectiveness (and cost-benefit) of a project administering deworming and weekly iron-folic acid supplementation to control anaemia in women of child-bearing age.	STH and weekly iron supplements	• Women of child-bearing age	• Anaemia cases averted	The cost per anaemia case averted was estimated to be US$4.24.	Same study
			• Village health workers	• A cost benefit ratio based on the labour market productivity for women of reproductive age after removal from anaemia	The benefit: cost ratio was estimated to be 6.70:1, i.e. for each dollar invested in the weekly iron supplementation and deworming program the monetary value in terms of productivity was US$6.70.	
			• Treatment every four months in the first year and every six months thereafter.			
[79]	Cost-effectiveness of school-based anthelmintic treatments against anaemia in children.	STH and Schistosomiasis	• SAC	• Anaemia cases averted	The cost per anaemia case averted by deworming school children was in the range of US$6–8.	[17]
			• School-based treatment			
			• Annual			
[31]	Comparison of mass, targeted and selective chemotherapy with levamisole for *Ascaris* control.	*Ascaris*	• *Varied (selective, targeted (to Pre-SAC and SAC), and mass)*	• Egg reduction per gram of faeces	The mass and targeted treatment strategies were considerably more cost-effective then selective treatment.	Same study
			• Mobile teams		Cost per 1000 egg reduction per gram of faeces:	
			• Three monthly		• Selective treatment: ₦5,004,	
					• Targeted treatment: ₦611,	
					• Mass treatment: ₦451.	
[26]	Cost-effectiveness of school-based and community distributed chemotherapy for schistosomiasis and STH control.	STH and Schistosomiasis	• SAC and targeted adults	• Infections averted	The estimated cost per infection averted in the treated population (children and adults) was US$2.50.	Same study
			• Combination			
			• Annual			
[32]	The cost-effectiveness of selective health interventions for the control of STH in rural Bangladesh.	STH	• *Varied (See Table* 1 *)*	• Reduction of mean egg counts	A single round of albendazole to all household members (over the 18 month study) was more cost-effective than chemotherapy to all household members followed by subsequent six monthly chemotherapy to all children. The two regimens involving health education were the least cost-effective.	Same study
			• Mobile teams	• Reduction in prevalence		
			• *Varied (one round over 18 months vs six monthly*)			
[73]	Cost-effectiveness (and cost-benefit) of school-based STH and Schistosomiasis control.	STH and Schistosomiasis	• SAC	• DALY	Treating SAC is highly cost-effective – US$5 per DALY averted (it was noted that this estimate ignores the indirect benefits for untreated children and adults in the treatment area). Though in areas without schistosomiasis, the cost per DALY averted was estimated to be US$280 – discussed in [42].	[16]
			• School-based treatment	• Additional years of school participation	The cost per additional year of school participation was estimated to be US$3.50 and deworming was found to increase the net present value of wages by over US$30 per treated child.	
			• Biannual albendazole (annual praziquantel)	• Net present value of wages		
[80]	Effects of the Zanzibar school-based deworming program on iron status of children.	STH and Schistosomiasis	• SAC	• Anaemia cases averted	The cost per moderate to severe anaemia case (Hb < 90 g/L) averted over one year (with four monthly mebendazole treatment) was US$3.57, increasing to US$16.30 for the cost per severe anaemia averted (<70 g/L).	Unpublished data
			• School-based treatment			
			• Four monthly			
*Modelling (type of model – see Box 2)*						
[48]	Cost-effectiveness of school-based *Ascaris* control (*dynamic model*).	*Ascaris*	• SAC	• DALY	The analysis indicates that treating SAC is highly cost-effective; US$8 per DALY averted (for a high prevalence community).	Unpublished data
			• School-based treatment			
			• Annual			
[21]	Cost-effectiveness analysis of mass anthelmintic treatment: effects of treatment frequency on *Ascaris* infection (*dynamic model*).	*Ascaris*	• Mass treatment (i.e. all three age groups)	• Unit reductions in mean worm burden	If the aim of an intervention is to reduce *Ascaris* related morbidity using mass treatment, then it is more cost-effective to intervene in higher transmission areas. Furthermore, relatively long intervals between treatments offer the most cost-effective strategy.	Unpublished data
			• Mobile teams	• Infection cases averted		
			• *Varied (between every four months and every two years)*	• Disease cases averted		
[44]	Options for chemotherapeutic control of *Ascaris* (*dynamic model*).	*Ascaris*	• *Varied (mass vs, targeted (to SAC and Pre-SAC))*	• Infection cases averted	Child-targeted treatment can be more cost-effective than mass treatment in reducing the number of disease cases. The results also imply that (with the assumed circumstances) enhancing coverage is more cost-effective than increasing frequency of treatment.	[21] – which was based on unpublished data
			• Mobile teams	• Disease cases averted		
			• *Varied (between every six months and every two years)*			
[45]	The cost-effectiveness of using different thresholds for determining the treatment frequency (*static distribution model*).	STH	• Pre-SAC and SAC	• Cost per infected person treated	This analysis suggests that a new three-tier treatment for deciding initial treatment frequency (if the combined prevalence is above 40 %, treat all children once a year; above 60 % treat twice a year; and above 80 % treat three times a year), would be more cost-effective than the current WHO recommended thresholds.	[16, 17, 22, 24]
			• Combination of school-based treatment and Child Health Days	• Cost per moderately/heavily infected person treated,		
			• *Varied at different thresholds*	• Cost per diseased person treated		
[47]	The potential cost-effectiveness of a hookworm vaccine (*static model*).	Hookworm	• SAC and non-pregnant women of child-bearing age	• DALY	A hookworm vaccine may provide not only cost savings, but potential health benefits to both SAC and non-pregnant women of child-bearing age. The most cost-effective strategy may be to combine vaccination with the current drug treatment.	[4, 39, 81]
			• Combination of school-based treatment and CDDs			
			• Annual			
*Policy documents/reports*						
[4]	Cost-effectiveness of school-based STH control.	STH ± Schistosomiasis	• SAC	• DALY	This analysis indicates that treating SAC is highly cost-effective; US$3.41 per DALY averted. (In combination with praziquantel to treat schistosomiasis this changes to US$8–19 per DALY averted.)	Not clearly stated
			• School-based treatment		*Though it should be acknowledged that this estimate was found contain a number of errors* [43]*. GiveWell re- estimated the cost-effectiveness (using a different methodology) and obtained US$30–$80 per DALY averted* [43]*.*	
			• Annual			
[34]	Cost-effectiveness of treating SAC for STH and schistosomiasis.	STH and Schistosomiasis	• SAC	• DALY	This analysis indicates that treating SAC is within the range of being considered highly cost-effective; US$6–33 per DALY averted.	Unpublished data
			• Mobile teams (via the school)			
			• Annual			
[41]	Cost-effectiveness of treating SAC.	Not clear	• SAC	• DALY	This analysis indicates that treating SAC is within the range of being considered highly cost-effective; US$15–30 per DALY averted.	Not clearly stated
			• Not clear			
			• Not clear			

More detailed information regarding the costs (when available) is provided in Table 1. CDDs; Community drug distributers, Pre-SAC; Pre-school aged children, SAC; School-aged children. School-based delivery systems were defined as those utilising teachers and other school officials (not just distributing the drugs at the school)

We identified only seven studies exploring the cost-effectiveness of alternative treatment strategies [21, 29–32, 44, 45]: four of which used mobile teams (Table 2). A summary of aim, method and primary conclusions of the identified studies is presented in Table 2 – which have generally been replaced by school/community-based delivery systems (Fig. 3).

It should be noted that for several of the reported cost-effectiveness estimates, the method of distribution and treatment frequency were not clearly stated and many did not employ any (or any detailed) sensitivity analysis. This lack of clarity, and at times poor quality, is not exclusive to STH, but has been found to be common in economic evaluations for parasitic diseases [46].

#### Adopted cost-effectiveness perspective

As with the cost studies discussed above, the clear majority of the cost-effectiveness studies were conducted from the perspective of the service provider i.e. the control programme (again this was rarely explicitly stated or justified). Only one cost-effectiveness analysis was found that used a societal perspective [47], and one the family’s (payer’s) perspective [29].

#### Choice of effectiveness measure

A variety of different effectiveness outputs were used for these analyses; such as egg reduction, infection cases averted, heavy cases averted and reduction in anaemia (Table 2). This variety in outcome metrics makes it difficult to compare the results of the different studies.

Due to the difficulties in developing statistical models linking the population dynamics of the STH to the incidence of different disease outcomes, most of the modelling studies used infection-based effectiveness metrics (i.e. reduction in mean worm burden (Table 2)). These studies defined disease as having a modelled worm burden above a certain threshold [21, 44, 45]. Only two modelling studies [47, 48] were found that used DALYs as the effect measure.

#### Sources of cost data

**Fig. 6:**
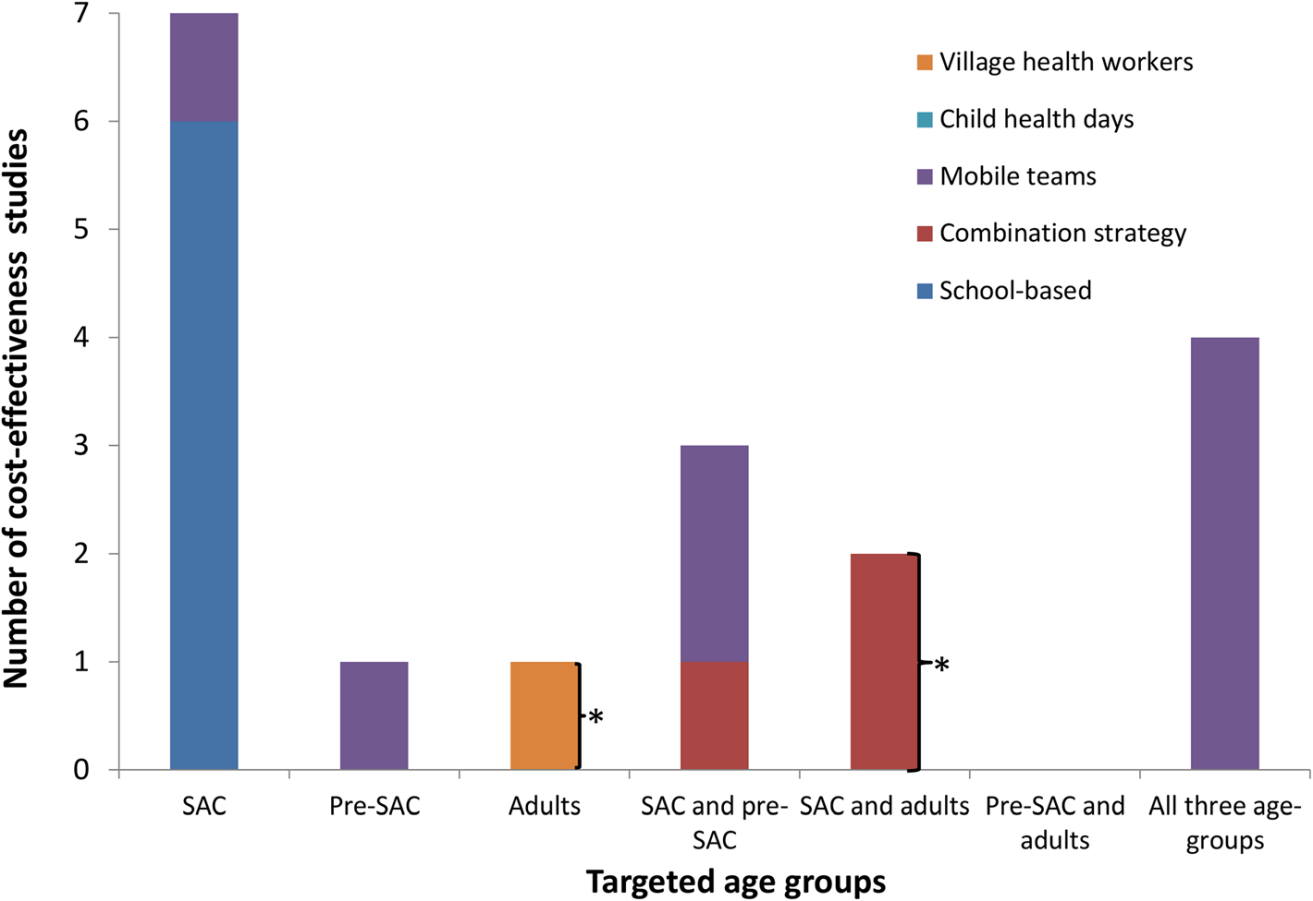
The number of identified cost-effectiveness estimates of STH control, stratified by target population and method of distribution. Pre-SAC; Pre-school aged children, SAC; School aged children. School-based delivery systems were defined as those utilising teachers and other school officials (not just distributing the drugs at the school). A combination strategy was defined as using both the school system and community drug distributers (CDDs). Some studies were counted more than once as the target population was varied within the study. *Targeted adults (such as those in at risk occupations)

We found that over a third of the identified cost-effectiveness analyses used costs associated with treatment distribution via mobile teams (Table 2 and Fig. 6), which have now mostly been replaced by school/community-based delivery systems (Fig. 3). Furthermore, many of the studies that did investigate school-based delivery programmes used costs (at least in part), based on the results of two Partnership for Childhood Development (PCD) studies [16, 17]. However, these results have been identified as potentially being artificially low because they did not include the external costs of the UK-based coordinating centre [39]. Several studies were found that used unpublished cost data (Table 2).

#### Type of method

Mathematical models can be particularly useful tools for investigating the cost-effectiveness of alternative STH treatment strategies and quantifying the impact of different epidemiological and programmatic settings on the generalisability of the conclusions. Furthermore, models can be used to make projections over long time horizons, capturing the long term benefits of interventions (empirical/data driven approaches using primary data from the field often have a limited time horizon of a few years and frequently only occur in one setting). However, only two studies [21, 44] were identified that investigated the cost-effectiveness of alternative STH treatment strategies using a dynamic model (the differences between dynamic and static models are defined Box 2) [49]. These studies only focused on *Ascaris*, and consequently the potential influence of the other STHs on the cost-effectiveness of different strategies was not explored. This is particularly important for hookworm, which has a notably different age-profile of infection than *Ascaris,* with the adults usually having a larger proportion of the overall worm burden [13]. Consequently, ignoring this aspect may underestimate the value of expanding MDA programmes to target the whole community. Furthermore, the costs used in these studies [21, 44] were based on the use of mobile teams (the main delivery method at the time) in Africa. The effect the use of the current school/community-based delivery systems has on the modelling conclusions has not been subsequently considered.

**Box 2: Model types**

**Table:** 

Static models: Static models are very widely used in economic evaluations but assume that the rate at which individual hosts acquire infection (the force of infection) is *uncoupled* from the abundance of infection in the population [49] i.e. they assume that an individual’s probability of being exposed to an infection is unaffected by an intervention (even if the abundance of infection is reduced) [50–52].
Dynamic models: Dynamic transmission models couple the rate of infection and the abundance of infection within the population (in this case eggs in the environment). Consequently, within these models the rate of infection changes if the level of infection is reduced due to an intervention [49–52]. These models therefore account for the fact that drug treatment programmes targeting STH will often not only benefit the individuals treated, but also reduce the risk of infection to others in the population (as the rate of transmission is reduced). This is particularly important to account for when investigating which age-groups should be targeted for treatment. It is also important to note that for the macroparasites (such as the STH) a dynamic model is also essential to account for the density dependent processes [53–56] which govern their transmission; such processes can lead to a highly non-linear impact of control against transmission [49].

## Discussion

### Limitations

A potential source of bias of the employed search strategy is that it would not retrieve cost and cost-effectiveness estimates that are not within the searched electronic databases (such as policy documents/reports etc.). To minimise this, references of the identified studies were searched and key members of the field were contacted regarding any unpublished data. Furthermore, it should be noted that the selection of studies was not performed in duplicate.

### Knowledge and research gaps

As our analysis of the published literature demonstrates, the quality and quantity of studies addressing the current policy decisions in STH control is low (Tables 1 and 2). We therefore highlight policy relevant areas which will require new research to address.

#### Costs of different treatment strategies

The majority of the identified costs were for programmes targeting only SAC (Fig. 2). There are very few studies reporting the costs associated with treatment strategies targeting multiple age groups (Pre-SAC, SAC and adults) within the context of the current school/community-based delivery systems. This is despite scaling up treatment for both Pre-SAC and SAC currently being the primary goal for STH control within the London Declaration on NTDs [6, 7]. Furthermore, in spite of biannual MDA being recommended by the WHO in areas with intense transmission/high prevalence [1], we found only two studies that reported the associated costs within a school-based programme (Fig. 4); both of which did not report the full economic costs [35, 36]. Detailed investigation of how the costs of increasing the treatment frequency change in different settings is vital, as it is unlikely to be simply double the cost of annual treatment [35, 36, 57, 58]. This lack of data is further highlighted when looking at the costs used in the identified cost-effectiveness analyses – with the clear majority of studies relating to interventions targeting only SAC and most of the few alternatives pertaining to the use of mobile teams (Fig. 6). However, the latter have now mostly been replaced by school/community-based delivery systems (Fig. 3).

Furthermore, a greater understanding of the economic value of the STH drug donation programmes, and how these may be influenced by any change in treatment strategy, is required.

#### Economies of scale

Comprehensive costing functions that can account for how the costs of STH treatment change with the number treated are essential for estimating the costs of scaling-up programmes (Fig. 5). Such economies of scale have also been reported by Goldman *et al.* [59] regarding the costs of MDA for LF control.

These economies of scale have been found to have significant implications when investigating the cost-effectiveness of STH control [Turner HC, Truscott, JE, Fleming, FM, Hollingsworth TD, Brooker SJ, and Anderson, RM: Is scaling up mass drug administration for the control of the soil-transmitted helminths cost-effective? Submitted] and need to be carefully considered when comparing the relative costs of different strategies (particularly between different studies/settings) [38]. Additionally, the relative increase in costs for adopting a new strategy (such as increasing the treatment frequency or expanding to target more age groups), will likely change depending on the scale of which it is adopted within a district. Understanding these interactions will be essential to best inform practical policy decisions [60].

#### Data collection and analysis methods

There is a growing need for standardised tools for costing data collection to allow for valid comparison between different studies.

Based on our analysis of the current STH costing studies (Table 1) and those presented in a review of the cost and cost-effectiveness of insecticide-treated nets [61], we present an outline of our recommendations for collecting and presenting STH treatment cost results in Table 3.

**Table 3:** Recommendations for collecting and presenting cost results (based on [61])

Perspective	• The perspective of the analysis (which determines whose costs are included) should be clearly stated and justification provided.
	• For STH treatment programmes the costs of accessing treatment are normally negligible and therefore we recommend the use of a service provider’s perspective. However, if other interventions are also used (such as WASH) which may incur patient level costs the use of a societal perspective should be considered.
Output	• The results should clearly state the treatment frequency, target age group(s), method(s) of distribution and the reported coverage (stratified by age groups and treatment method).
	• For cost-effectiveness studies, the effectiveness metric(s) (such as cost per child treated, cost per health outcome averted) should be clearly stated and justified.
Resource identification	• Include the economic value of the time volunteered by teachers/community drug distributors (CDDs) and donated items: their time should be valued as the equivalent to their occupation had they not been volunteering calculated using local pay scales.
	• Exclude research costs.
	• Include relevant overheads of collaborating organizations (e.g. non-governmental organizations (NGO) contributions).
	• Clarify what management capacity is assumed to exist and whether the study is calculating an average cost of a programme or an incremental cost of adding an additional intervention within existing programme.
Resource measurement and valuation	• Where appropriate, account for integrated NTD control activities and shared resources between other control programmes, thereby indicating economies of scope.
	• For all capital items a discount rate of 3 % should be applied-to be consistent with the rate used by the World Bank [41]. This use of different discount rates (such as country-specific estimates) should be explored in the sensitivity analysis.
Sensitivity analysis	• To reflect the uncertainty in measurements a sensitivity analysis should be carried out on the main factors, including: discount rate, useful life of capital items, staff costs, fuel costs, and method used to value volunteers’ time.
	• Where it is necessary to estimate a share of resources contributed from other programmes or interventions (particularly in the context of integrated NTD control), the assumptions used should be subjected to sensitivity analysis.
Reporting of results	• Cost estimates should be provided in US$ and adjusted for inflation.
	• The cost year and exchange rates should be clearly stated.
	• Clearly state whether costs are per treatment round or costs per year.
	• Clearly state how the drugs were distributed (i.e. through schools by teachers and/or by CDD) and the number treated by each method stratified by age and school enrolment status (i.e. indicate how may school-aged children were treated by the CDD).
	• Where possible indicate which costs were fixed and which variable.
	• Provide costs stratified by individual programme activities (e.g. programme running costs, community sensitization, training, drug distribution and treatment, monitoring and evaluation).
	• Provide costs stratified by resource type (e.g. personal, equipment, supplies, transportation and facilities).
	• Report both the per capita total cost per treatment and delivery cost per treatment (as well as drug costs).
	• Report the economic cost both with and without the value of donated drugs.
	• Report the number treated each round (and coverage).
	• When investigating more than one control strategy, details of how the costs/values of different programmatic activates were different should be provided and how shared costs have been attributed.

In particular, clarity and consistency in the methods used to handle the pooled costs of shared activities between different strategies/other control programmes is needed, particularly as NTD control programmes become more integrated. It is important that any adjustments to the data are made clear to allow for valid comparison between different studies, especially if these costs are to be incorporated into any cost-effectiveness analysis. Furthermore, there is variation in the methods used to apply an economic value to the donated time of community volunteers and teachers for NTD control [57].

Standardised costing collection and analysis methods will be a crucial step in identifying the real underlying drivers of variation in treatment costs.

#### Understanding drivers of variation in delivery costs

Since it would be impossible to run research studies in every setting, it is essential the field gains a more general understanding of the factors that drive the variation in MDA delivery costs between different countries/health systems. For example, the STH control programmes in Asia were more likely to use a “sibling approach” to reach un-enrolled SAC [62], whereas the African programmes were more likely to use CDDs. This may influence the method, and hence the cost, as well as the achieved coverage of expanding programmes to target Pre-SAC and/or adults. Furthermore, a school-based programme that has a relatively high delivery cost, may have a lower incremental cost of expanding to incorporate adults due to the baseline investment in training and recruitment. Understanding how the structure of different health systems may influence the relative costs of adopting different strategies will be vital to further investigate what the most cost-effective control strategies are in different areas.

Sensitivity analyses must be performed in any modelling study in order to investigate the robustness and generalisability of the conclusions to other settings. As part of this, the structure of the health systems and the potential difference in the relative costs of new strategies needs to be considered in economic modelling studies. This is currently almost never done for STH control, yet may be very influential in terms of what the optimum strategy is in a given area and the generalisability of modelling conclusions. This further highlights the need for widespread costing studies in a range of areas and the investigation of the key drivers of the costs.

#### Consequences of integration

A notable research gap is the lack of understanding regarding the costs of integrated NTD control [63, 64] and how integration may influence the costs of implementing different control strategies (economies of scope).

Furthermore, the implications for STH of LF-related MDA being halted, leaving these areas at a potentially increased risk of STH recrudescence need to be evaluated. In contrast, the potential additional benefit of biannual MDA, which is being considered for LF control [58], could have large benefits for STH which are yet to be evaluated.

#### Effectiveness metrics

A notable research gap is the lack of clearly defined metrics to evaluate the impact of STH interventions, particular in terms of morbidity [65]. At present the easily measurable metrics in the field are the intensity and prevalence of infection (both measured indirectly by egg output in faeces). However, the level of egg output per worm can vary in different areas around the world [53], highlighting the difficulty in translating number of eggs to the number of worms.

A debate is needed amongst the NTD research field regarding what effectiveness measure is best, and feasible to acquire in quantitative studies. The imprimatur of WHO in such debates to define the best metrics is desirable. This would allow future studies to adopt a common metric/design structure. It will also be important to consider how different programmatic aims (such as morbidity control versus reductions in transmission), may require different effectiveness metrics, as this will affect the optimum strategy [66].

More research is needed to develop study designs and statistical methods that relate the disease burden of STH to experience of infection [67], and to link these disease frameworks into dynamic transmission models. However, the relationship between STH infections and disease is complex, and likely depends on a number of host specific factors – such as age, time infected, and underlying nutritional status [68]. Consequently, thresholds of infection intensify above which individuals are said to suffer from disease (as used as an effectiveness measure in some modelling studies), need to be treated with caution.

DALYs are often used to measure the overall burden of a particular disease (and are the primary metric used by the global burden of disease studies [69]). However, the design of the DALY contains inherent flaws that fail to acknowledge the implications of context on the burden of disease for the poor [70]. This results in the significant underestimation of the disability weights for the NTDs, which are most prevalent in poor populations [70]. There is a growing need for the further development of more comprehensive disability metrics for NTDs (such as the quality-adjusted life year (QALY)) which can more effectively capture the disease burden of STH infections and better evaluate the cost-effectiveness of different control strategies [71, 72]. Any studies using DALYs or QALYs as their effectiveness metric should clearly describe the methodology used to estimate them (and highlight the potential limitations and uncertainties of the estimates).

It is also important to consider the non-health related benefits of deworming [60] – such as on education, and capital development (as discussed by Miguel and Kremer [73]).

## Conclusions

In this time of rapidly expanding MDA coverage and the new commitments for STH control it is essential that resources are allocated in an efficient manner to have the greatest impact. The majority of the identified cost-effectiveness analyses for STH treatment pertained to programmes targeting only SAC, with relatively few studies exploring the cost or cost-effectiveness of alternative strategies. The optimum treatment strategy in terms of targeting of different age-groups, or frequency of treatment, has been shown to be highly specific to the local epidemiology [8–10]. Consequently, in some areas (particularly those with high transmission) it may be more cost-effective or even cost saving to initially use more expensive but intensive interventions—such as expanding to treat other age groups—in order to reduce programme duration and the overall net cost of the control. Detailed and accurate costs of targeting different combinations of age groups or increasing the treatment frequency will be essential to permit further evaluation of the most cost-effective control strategies.

Due to the inconsistences in the collection/analysis methods used in published STH costing studies, it is not possible to draw firm conclusions regarding the relative costs of the different strategies. This inconsistency, coupled with a significant lack of data, constitutes a major research gap in for the optimization of STH control. However, there are numerous opportunities to collect these data as programmes are scaled-up, and whilst lymphatic filariasis programmes are still operating at scale. With suitable guidance, countries could collect locally relevant information which could guide the long-term investment in these programmes over the coming years. We present an outline of our recommendations for collecting and presenting STH treatment cost results in Table 3.

## Additional files

Additional file 1:
**Search terms for Pubmed.**
Additional file 2:
**PRISMA 2009 Checklist.**

## Abbreviations

CDDs: Community drug distributers
DALY: Disability-adjusted life year
GNI: Gross National Income
LF: Lymphatic filariasis
MDA: Mass drug administration
NTD: Neglected tropical diseases
PCD: Partnership for Childhood Development
Pre-SAC: Pre-school children
PCT: Preventive chemotherapy
QALY: Quality-adjusted life year
SAC: School-aged children
STH: Soil-transmitted helminths
WHO: World Health Organization
