# Supplementary material for: Cost and cost-effectiveness of soil-transmitted helminth treatment programmes: systematic review and research needs
Source: Parasit Vectors. 2015 Jul 3;8:355. doi: 10.1186/s13071-015-0885-3 (PMC4499443; doi:10.1186/s13071-015-0885-3)
Supplement: Additional file 1: — Search terms for Pubmed. [file 13071_2015_885_MOESM1_ESM.docx]

**Supporting Information: Search terms for Pubmed**

(Soil-transmitted[All Fields] AND ("helminths"[MeSH Terms] OR "helminths"[All Fields] OR "helminth"[All Fields]) OR “Helminthiasis"[All Fields] OR “intestinal parasites"[All Fields] OR “intestinal helminths” [All Fields] OR “Ascaris” [All Fields] OR “Trichuris” [All Fields] OR “hookworm” [All Fields]) and ("Helminthiasis/economics"[MAJR] OR "Benzimidazoles/economics"[MAJR] OR "Albendazole/economics"[MeSH Terms] OR "Anthelmintics/economics"[MeSH Terms] OR “Cost"[All Fields] OR “Cost analysis” [All Fields] OR "economics"[Subheading] OR "economics"[ MeSH Terms] OR "economic"[All Fields] OR "cost-benefit analysis"[MeSH Terms] OR ("cost-benefit"[All Fields] AND "analysis"[All Fields]) OR "cost-benefit analysis"[All Fields] OR ("cost"[All Fields] AND "effectiveness"[All Fields]) OR "cost effectiveness"[All Fields])
